# Optimizing Low-Concentration Mercury Removal from Aqueous Solutions by Reduced Graphene Oxide-Supported Fe_3_O_4_ Composites with the Aid of an Artificial Neural Network and Genetic Algorithm

**DOI:** 10.3390/ma10111279

**Published:** 2017-11-07

**Authors:** Rensheng Cao, Mingyi Fan, Jiwei Hu, Wenqian Ruan, Kangning Xiong, Xionghui Wei

**Affiliations:** 1 Guizhou Provincial Key Laboratory for Information Systems of Mountainous Areas and Protection of Ecological Environment, Guizhou Normal University, Guiyang 550001, China; 18230825324@163.com (R.C.); fanmingyifmy@163.com (M.F.); wenqianruan@yahoo.com (W.R.); 2 Cultivation Base of Guizhou National Key Laboratory of Mountainous Karst Eco-Environment, Guizhou Normal University, Guiyang 550001, China; xiongkn@163.com; 3 Department of Applied Chemistry, College of Chemistry and Molecular Engineering, Peking University, Beijing 100871, China; xhwei@pku.edu.cn

**Keywords:** water treatment, mercury, artificial neural networks, genetic algorithm, artificial intelligence

## Abstract

Reduced graphene oxide-supported Fe_3_O_4_ (Fe_3_O_4_/rGO) composites were applied in this study to remove low-concentration mercury from aqueous solutions with the aid of an artificial neural network (ANN) modeling and genetic algorithm (GA) optimization. The Fe_3_O_4_/rGO composites were prepared by the solvothermal method and characterized by X-ray diffraction (XRD), transmission electron microscopy (TEM), atomic force microscopy (AFM), N_2_-sorption, X-ray photoelectron spectroscopy (XPS), Fourier transform infrared spectroscopy (FTIR) and superconduction quantum interference device (SQUID). Response surface methodology (RSM) and ANN were employed to model the effects of different operating conditions (temperature, initial pH, initial Hg ion concentration and contact time) on the removal of the low-concentration mercury from aqueous solutions by the Fe_3_O_4_/rGO composites. The ANN-GA model results (with a prediction error below 5%) show better agreement with the experimental data than the RSM model results (with a prediction error below 10%). The removal process of the low-concentration mercury obeyed the Freudlich isotherm and the pseudo-second-order kinetic model. In addition, a regeneration experiment of the Fe_3_O_4_/rGO composites demonstrated that these composites can be reused for the removal of low-concentration mercury from aqueous solutions.

## 1. Introduction

Heavy metals are a group of metallic elements (e.g., Hg, Cr, Cd and Pb) that are of a high gravity and cannot be degraded by living organisms [1,2]. Different heavy metals have been found in various sources, e.g., industrial effluents, agricultural and domestic wastes [3,4,5,6]. These heavy metals can accumulate in living organisms and can thus cause a serious risk to human health [4,7]. Among these heavy metals, mercury has aroused a special attention, and has been included on the list of priority pollutants by the United States Environmental Protection Agency (US EPA) and the European Union (EU). The major effects of mercury poisoning manifest in that it can easily pass the blood-brain barrier and affect the fetal brain. The symptoms of Hg poisoning not only cause neural disorders, but also exert damage on the cardiovascular system, kidney, bones, etc. [8]. Therefore, the removal of mercury from wastewater is of pivotal importance because of its strong negative impacts on organisms.

If primary treatment (primary settling tank and grit chamber, grid) and secondary treatment (biological treatment) cannot completely remove pollutants from wastewater [9], tertiary treatment (or advanced treatment) of wastewater with low concentrations of pollutants can be carried out by using a range of physico-chemical processes, such as ozonation, Fenton reagent oxidation, ion exchange, chemical precipitation, membrane separation, coagulation, flocculation and active carbon adsorption [10]. Among them, adsorption has proven to be a convenient and effective method due to its low initial cost, flexibility in design, easy operation, and insensitivity to biological materials in an aqueous environment [11]. Iron and iron oxide have been considered to be excellent adsorbents, with a higher efficiency for the removal of various heavy metal ions, such as Hg(II), Cr(VI), Ni(II), Cu(II), Cd(II) and Pb(II) [3,8,12,13,14,15]. These adsorbents have been widely used as heavy metal ion removers owing to their strong superparamagnetism, low toxicity and facile preparation process [14].

Fe_3_O_4_ nanoparticles, as a magnetic material, exhibit a finite-size effect or a high specific surface area, resulting in a higher adsorption capacity for heavy metal removal. In addition, the rapid separation of metal-loaded magnetic adsorbent from a solution can be achieved using an external magnetic field. Thus, an efficient, economic, scalable and non-toxic synthesis of Fe_3_O_4_ nanoparticles is highly preferred for fundamental research and potential applications [3,16,17]. However, the fast oxidation and rapid agglomeration have been the major challenges when using these nanomaterials. In order to solve these problems and prevent the side pollution caused by the released nanomaterials, it has been reported that these nanoparticles were supported on some inert materials. As a single layer for sp^2^-bonded carbon atoms patterned in a hexagonal lattice [18], graphene exhibits prominent thermal stability, superior electronic conductivity, remarkable structural flexibility, a large surface area and abundant surface groups. Therefore, graphene can be adopted to immobilize and well-disperse the nanoparticles [16,19] and has already revealed a great number of potential environmental applications [20,21]. Fe adsorption on graphene has previously been studied with computational simulations [22,23,24,25,26]. The adsorption of 15 different transition metal adatoms (including Fe) on graphene was studied using the first-principles density-functional theory with generalized gradient approximation. This shows that the adsorption is characterized by a strong hybridization between the adatom and graphene electronic states. The favored adsorption site indicates the main chemical bond between the adsorbate and graphene [22]. The density-of-states calculations and charge density contour plots reveal a charge transfer from the iron s orbitals to the d orbitals. Adsorbed iron atoms covalently bind to the graphene substrate, verified by the strong hybridization of iron d-states with the graphene bands in the energy region just below the Fermi level [25]. The graphene-based materials have become a focus for many researchers, especially for the removal of hazardous heavy metal ions from polluted water [27].

Chemical methods in water treatment can offer potentially low cost and large-scale production of graphene-based hybrid materials. Most recently, it has been reported that graphene oxide (GO) and magnetite iron oxide nanoparticles (Fe_3_O_4_ NPs) can adsorb and remove dyes [21,28,29,30,31,32,33], polychlorinated biphenyl [34], imide fungicides [35], aniline and p-chloroaniline, etc. [36]. Heavy metal removal in water treatment is a complex process due to the interaction of variables and the nonlinear behavior of these processes. As a result, determination of the optimum experimental conditions is extremely important to obtain maximum efficiency. Modelling of such a process can facilitate studies of the influence of each parameter in order to predict removal efficiency with fewer experimental runs [37]. Artificial intelligence tools (e.g., artificial neural networks (ANNs), genetic algorithm (GA), support vector machine (SVM), simulated annealing (SA), and Monte Carlo simulation (MCS)) have been widely applied in various fields, e.g., autonomous driving, big data, pattern recognition, intelligent internet search, image understanding, automatic programming, robotics and human-computer games. These tools, in combination with such approaches as orthogonal design, response surface methodology (RSM) and uniform design, can efficaciously optimize the processes of pollutants removal [38]. ANN was inspired by biological neurons and derived from artificial intelligence (AI) research, which can well describe multivariate nonlinear problems with suitable amounts of data and appropriate training algorithms applied [39]. GA can avoid local optima frequently by promoting exploration of the search space and thus can be applied to solve a variety of optimization problems, including those in which the objective functions are discontinuous, non-differentiable, stochastic or highly nonlinear [40].

The objective of this study was to apply reduced graphene oxide-supported Fe_3_O_4_ (Fe_3_O_4_/rGO) composites for low-concentration mercury removal from aqueous solutions. The Fe_3_O_4_/rGO composites were synthesized by the solvothermal method and subsequently characterized via different techniques, such as X-ray diffraction (XRD), transmission electron microscopy (TEM), atomic force microscopy (AFM), N_2_-sorption, X-ray photoelectron spectroscopy (XPS), Fourier transform infrared spectroscopy (FTIR) and a superconduction quantum interference device (SQUID). Then, RSM and ANN-GA were employed to model and optimize the low-concentration mercury removal from aqueous solutions by the Fe_3_O_4_/rGO composites, and a comparison between the ANN-GA and RSM models was made for estimating their performance in the advanced water treatment process. Finally, the experimental data obtained were fitted to the adsorption isotherms (Langmuir and Freundlich) and the removal kinetics (pseudo-first-order and pseudo-second-order) and the regeneration of the Fe_3_O_4_/rGO composites as a sorbent were also investigated in this study.

## 2. Experiment

### 2.1. Materials

A stock Hg standard solution (GSB 04-1729-2004) was supplied by the National Center of Analysis and Testing for Nonferrous Metals and Electronic Materials in China. Graphite powder (particle size < 30 μm, purity > 99.85%) was purchased from Sinopharm Chemical Reagent (Beijing, China), ethylene glycol (EG) from Tianjin Fuyu Fine Chemical Co., Ltd. (Tianjin, China), sodium acetate (NaAc) from Tianjin Kemiou Chemical Reagent Co., Ltd. (Tianjin, China), hexahydrate ferric chloride (FeCl_3_·6H_2_O) from Chengdu Jinshan Chemical Reagent Co., Ltd. (Chengdu, China), hydrogen peroxide (H_2_O_2_, 30%) from Baiyin Liangyou Chemicals Reagent Co. Ltd. (Baiyin, China), potassium permanganate (KMnO_4_) from Tianjin Bodi Chemical Reagent Company (Tianjin, China), and concentrated sulfuric acid (H_2_SO_4_) from Beijing Chemical Factory (Beijing, China). All chemicals used in this study were of commercially available analytical grade. Ultrapure water was used throughout the experiment.

### 2.2. Preparation of the Fe_3_O_4_/rGO Composites

**Figure 1:**
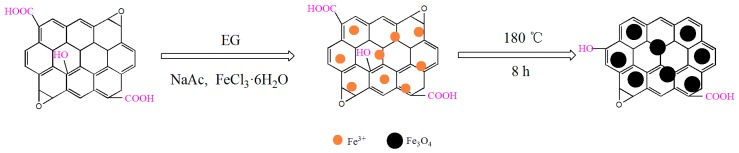
Synthesis process of the Fe_3_O_4_/rGO composites.

Graphene oxide (GO) was prepared by the modified Hummers method [41] and the obtained GO sheet (0.5 g) was exfoliated by ultrasonication in 80 mL of EG solution for more than 3 h (the water bath sonication conditions were 25 °C and 160 W). Then 1.6 g of FeCl_3_·6H_2_O and 3.2 g of NaAc were dissolved in the GO-EG solution at ambient temperature. After stirring for about 30 min, the solution was transferred into a 100 mL Teflon stainless-steel autoclave and kept at 180 °C for 8 h, followed by cooling to ambient temperature naturally. The black precipitate was centrifuged, washed with ethanol several times, and finally dried at 60 °C in a vacuum oven [11,29]. The synthesis process of the Fe_3_O_4_/rGO composites is shown in Figure 1 [42,43]. The preparation procedure of Fe_3_O_4_/GO composites is given in the Supplementary Materials.

### 2.3. Characterization of the Fe_3_O_4_/rGO Composites Synthesized

XRD measurements were recorded on a D8 Advance using the LynxEye array detector with a Cu-Kα X-ray source (generator tension = 40 kV, current 40 mA, Bruker Corporation, Karlsruhe, Germany). TEM images were taken by a TecnaiG2 F20 (FEI Co., Ltd., Hillsboro, OR, USA) microscope and the AFM images were recorded with a scanning probe microscope (BY2000-140610A, Being Nano-Instruments Ltd., Beijing, China) in tapping mode. XPS measurements were recorded on an ESCALAB 250Xi spectrometer using monochromatized Al Kα radiation (hν = 1486.6 eV), all binding energies were calibrated by using the contaminant carbon (C1S = 284.8 eV) as a reference (Thermo Electron Corporation, Waltham, MA, USA). FTIR measurement used a Nicolet 6700 spectrometer (Nicolet Instrument Corporation, Madison, WI, USA). Magnetization measurements were carried out using a SQUID magnetometer (MPMS XL-7, Quantum Design, Inc., San Diego, CA, USA) under applied magnetic field at room temperature. Brunner-Emmet-Teller (BET) surface areas of the Fe_3_O_4_/rGO composites were obtained from N_2_ adsorption isotherms at 77 K with a micromeritics 3 Flex surface characterization analyzer (outgass time: 3.0 h, outgass temperatue: 300.0 °C, Micromeritics Instrument Corporation, Norcross, GA, USA).

### 2.4. Removal of the Low-Concentration Mercury

Removal of the low-concentration mercury by the Fe_3_O_4_/rGO composites was performed in 100 mL centrifugal tubes. 20 mg of the Fe_3_O_4_/rGO composites was added to 50 mL of aqueous sample solution, which was shaken at 200 rpm for desired temperature and contact time. Then the composites were removed by a magnet and the concentrations of metal ions in the solutions were determined by an atomic fluorescence spectrometer (AFS-933, Beijing Titan Instruments Co., Ltd., Beijing, China). The removal efficiency (*E*) and removal quantity (*q_e_*) were calculated with the following Equations:

(1) $$E=\frac{C_{0}-C_{e}}{C_{e}}\times 100\%$$

(2) $$q_{e}=\frac{(C_{0}-C_{e})\times V}{m}$$

where *q_e_* is the removal quantity of the Hg ions per unit mass of adsorbent (µg·g^−1^), *C*_0_ and *C_e_* are the initial and equilibrium concentrations of the Hg ions (µg·L^−1^), *V* is the volume of the solution (L), and *m* is the dry weight of the Fe_3_O_4_/rGO composites (g).

The regeneration of adsorbents is highly crucial in practical applications. A good adsorbent should possess a high adsorption capability as well as a good desorption property [44,45]. Therefore, the regeneration of the adsorbent was carried out in this study as follows. 20 mg of Fe_3_O_4_/rGO composite was added to a flask containing 50 mL of the Hg ion solution (10 µg/L) at initial pH = 7 and kept on a shaker for 70 min. After removal, the composites were separated using an external magnet and washed with 80 mL of 0.01 M HCl and deionized water several times at room temperature, then the black precipitate obtained was dried at 40 °C in a vacuum oven for 12 h. The regeneration efficiency (RE) corresponds to the ratio between the adsorption capacity of a given cycle and that of the original nanocomposites. The step stripping efficiency (SSE) is defined as the ratio between the adsorption capacity of a given cycle and that of the previous one [44].

### 2.5. RSM Method

The Box-Behnken design (BBD) is a commonly used RSM method that is a collection of mathematical and statistical techniques for the modeling and analysis of problems in which a response of the interest is influenced by several variables [46]. In the present study, BBD was employed to investigate the effects of different operating factors on the low-concentration mercury removal capacity, reveal the optimum conditions for the mercury removal, and build models [47]. The BBD model was established based on the Design-Expert software version 8.0.6 (Stat-Ease, Inc., Minneapolis, MN, USA) for the optimization of removal process. The experimental design was applied after the range of each variable (maximum and the minimum) was selected as shown in Table S1. Generally, the mathematical relationship between the response *Y* (removal capacity of the Hg ions) and these variables can be described by the following second-order polynomial Equation [9]:

(3) $$\begin{array}{l} Y=\beta_{0}+\beta_{1}x_{1}+\beta_{2}x_{2}+\beta_{3}x_{3}+\beta_{4}x_{4}+\beta_{12}x_{1}x_{2}+\beta_{13}x_{1}x_{3}+\beta_{14}x_{1}x_{4}+\beta_{23}x_{2}x_{3}+\beta_{24}x_{2}x_{4}+\beta_{34}x_{3}x_{4}+\beta_{11}{x_{1}}^{2}+\beta_{22}{x_{2}}^{2}+\\ \beta_{33}{x_{3}}^{2}+\beta_{44}{x_{4}}^{2} \end{array}$$

where *Y* represents the removal percentage of the Hg ions, *x*_1_, *x*_2_, *x*_3_ and *x*_4_ represent independent variables, and *β*_0_ is a constant offset term, *β*_1_, *β*_2_, *β*_3_ and *β*_4_ are linear coefficients. However, *β*_12_, *β*_13_, *β*_14_, *β*_23_, *β*_24_, and *β*_34_ are interaction coefficients, and *β*_11_, *β*_22_, *β*_33_ and *β*_44_ represent the quadratic coefficients which are computed from the predicted responses.

The validity of the equation was analyzed by using ANOVA (analysis-of-variance), and fit quality of the equation was judged from the coefficients of correlation and *p*-value.

### 2.6. ANN-GA-Based Modeling and Optimization for the Removal of the Low-Concentration Mercury

**Figure 2:**
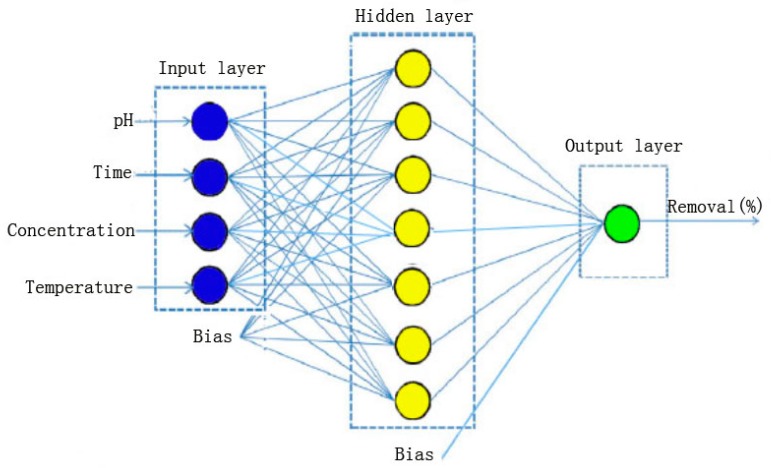
Structure of a back-propagation artificial neural network.

ANN is a highly simplified model of the structure for biological neural systems. The fundamental processing element of ANN is an artificial neuron (or simply a neuron). A biological neuron receives inputs from other sources, combines them, performs generally a nonlinear operation on the result, and then outputs the final result [38]. An ANN was applied to build the predictive model in this study, which consists of an input layer, one hidden layer and an output layer [38] as shown in Figure 2. The inputs for the network are temperature, initial pH, initial Hg ion concentration and contact time for the Hg ions; the output is the percentage of the Hg ion removal. The connections between the inputs, hidden and output layers consist of weights and biases that are considered as the parameters of the neural network.

The data obtained from the experimental values for the removal percentage of the low-concentration mercury was applied for network training to construct a network model that could compute the predicted removal percentage values from the inputs using the MATLAB 2005a software (MATLAB, Natick, MA, USA). All experimental data were divided randomly into two groups (24 data sets for training and five data sets for testing). The data (input and output) for the ANN models were normalized between −1 and 1 to avoid numerical overflows due to very large or small weights. The normalization equation applied was as follows [48]:

(4) $$j=2\times \frac{x_{i}-x_{\min}}{x_{\max}-x_{\min}}-1$$

where *j* is the normalized value of *x_i_*, the *x*_max_ and *x*_min_ are the maximum and minimum value of *x_i_*, respectively.

The results of various network structure and training procedures were compared based on the mean squared error (*MSE*) and the coefficient of determination (*R*^2^), which can be defined as follows [49]:

(5) $$MSE=\frac{1}{N}\sum_{i=1}^{N}{\left(y_{prd,i}-y_{exp,i}\right)}^{2}$$

(6) $$R^{2}=1-\frac{\sum_{i=1}^{N}\left(y_{prd,i}-y_{exp,i}\right)}{\sum_{i=1}^{N}\left(y_{prd,i}-y_{m}\right)}$$

where *y_prd,i_* is the predicted value by the ANN model, *y_exp,i_* is the experimental value, *N* is the number of data, and *y_m_* is the average of the experimental value.

A GA is inspired by the process of natural selection and genetic evolution and has been proven to be a successful method for solving a variety of optimization problems, applying mutation and crossover to a population of encoded variable spaces. The algorithm explores different areas of the parameter space, and directs the search to the region where a high probability of a global optimum exists. GA based optimization processes can be executed using trained ANN models as the fitness functions to give the global optimized solutions [40]. In the proposed method, after the ANN is trained, GA can be used to optimize the input variables with the objective of maximizing the percentage of low-concentration mercury removal.

## 3. Results and Discussion

### 3.1. Characterization of the Fe_3_O_4_/rGO Composites

XRD patterns were obtained for GO, the Fe_3_O_4_/rGO composites and Fe_3_O_4_/GO composites (Figure S1). A sharp peak at around 2θ = 10.8° is attributed to the (002) crystalline plane of GO (Figure S1a) [50]. The peaks at 2θ values of 30.1° (220), 35.28° (311), 43.2° (400), 56.9° (511) and 62.6° (440) are consistent with the standard XRD data for the cubic phase Fe_3_O_4_ [29] in Figure S1b, thus the XRD pattern confirms the successful preparation of the Fe_3_O_4_ particles in the composites by using the solvothermal method. Furthermore, an extra characteristic peak of GO (2θ = 10.8°) was found in the patterns of Fe_3_O_4_/GO composites compared with that of the Fe_3_O_4_/rGO composites (Figure S1c).

As displayed in the representative TEM images of the Fe_3_O_4_/rGO composites (Figure S2a), the Fe_3_O_4_ particles were anchored on the surface of rGO sheets with a diameter of ca. 300 nm, and no obvious aggregation of Fe_3_O_4_ on rGO was observed. As shown in Figure S2d, the size of the Fe_3_O_4_/rGO composites is larger than that of the Fe_3_O_4_/GO composites. The topography image obtained by means of AFM for the Fe_3_O_4_/rGO composites (Figure S2b) further demonstrates that the Fe_3_O_4_ particles are dispersed and anchored onto the rGO. Meanwhile, the cross-section analysis of the AFM image indicates the heights of 2.1–121.6 nm for the Fe_3_O_4_/rGO composites and the existence of layer-by-layer structured rGO sheets in the Fe_3_O_4_/rGO composites. The low surface roughness (34.9 nm) was given by the root mean square (RMS) value of the topographic data for the composites [51], which is not beneficial for the adsorption capacity of the adsorbent.

As shown in Figure S3a, a hysteresis loop between the relative pressure 0.47 and 0.98 appears in the N_2_ sorption isotherm of the Fe_3_O_4_/rGO composites, which is considered to be a type IV curve that shows the presence of mesopores. Based on the isotherm, the BET specific surface area was calculated to 42.570 m^2^/g for the prepared Fe_3_O_4_/rGO composites. According to Figure S3b, the pore size distribution curve shows that the Fe_3_O_4_/rGO composites possess one kind of mesopore with sizes centered at 0–13 nm.

The chemical state of the elements was examined based on the XPS spectra obtained in this study for the Fe_3_O_4_/rGO composites. The broad peaks at around 710.9 eV and 724.5 eV are typical for the Fe2p, corresponding to the Fe2p_3/2_ and Fe2p_1/2_ spin orbit peaks of Fe_3_O_4_ [18], respectively (as shown in Figure S4b), implying the formation of a mixed oxide of Fe(II) and Fe(III). As shown in Figure S4e, XPS spectra of the Fe_3_O_4_/GO composites are similar to those of the Fe_3_O_4_/rGO composites. The C1s peak is well decomposable into three spectral components [29], which are sp^2^ C=C (at 284.8 eV), carbons in C–OH (at 286.7 eV) and carboxyl/epoxy C=O (at 288.4 eV) [11], respectively. The spectral decomposition of the Fe_3_O_4_/rGO composites (Figure S4d) reveals that the O1s peak can be generally considered as a joint contribution of the anionic oxygen in Fe–O (at 530.3 eV), the carbonyl oxygen in C=O (at 531.5 eV), and the oxygen in C–O (at 533.1 eV) [28]. The O, C and Fe contents in the Fe_3_O_4_/rGO composites are shown in Table S3.

The FTIR spectra of GO and the Fe_3_O_4_/rGO composites (Figure S5) show that in the spectrum of GO, the strong absorption at 3412 cm^−1^ is attributed to the O–H stretching vibration. The absorptions at 1728 cm^−1^ and 1624 cm^−1^ belong to the stretching vibrations of C=O and aromatic C=C. The C–O stretching vibrations of epoxy group and alkoxy are observed at 1220 cm^−1^ and 1062 cm^−1^, respectively. In the spectrum of the Fe_3_O_4_/rGO composites, a sharp peak around 580 cm^−1^ attributed to the Fe–O vibration of Fe_3_O_4_ is observed. This proves that the surface of the rGO sheets was effectively decorated by Fe_3_O_4_ particles [41].

The magnetization hysteresis loop of the Fe_3_O_4_/rGO composites at room temperature (Figure S6a) demonstrates that the resulting composites exhibit the characteristics of ferromagnetic materials with a saturation magnetization of 49.55 emu·g^−1^, which was slightly lower than that of the Fe_3_O_4_/GO composites (Figure S6b). The magnetism for these two kinds of composites was strong enough to ensure the convenient magnetic separation. Furthermore, as seen in the inset, the Fe_3_O_4_/rGO composites could homogeneously disperse in aqueous solutions and a magnet would attract the magnetic adsorbent to the wall of the vessel conveniently.

### 3.2. RSM Analysis

According to the BBD results, the response function with determined coefficients for the removal of the Hg ions in this study is given below:

(7) $$\begin{array}{l} Y=79.63-0.89x_{1}-0.53x_{2}-4.85x_{3}+5.79x_{4}-0.13x_{1}x_{2}-0.99x_{1}x_{3}+0.59x_{1}x_{4}+1.32x_{2}x_{3}-1.52x_{2}x_{4}+0.10x_{3}x_{4}\\ +0.43{x_{1}}^{2}+0.45{x_{2}}^{2}-0.14{x_{3}}^{2}+0.81{x_{4}}^{2} \end{array}$$

The mutual interactive effects of the combination of independent variables on the low-concentration mercury removal efficiency in the nonlinear nature of 3 dimensions (3D) response surface plots (Figure S7) could demonstrate that there are interactions between each of the independent variables and dependent variable. As shown in Figures S7a,e,f, the Hg ion removal efficiency increased with an increase in initial pH from 8 to 10, which indicates that the alkaline conditions are conducive to the removal of the Hg ions. This phenomenon might be attributable to the active sites on the Fe_3_O_4_/rGO composites occupied by H^+^ under highly acidic conditions. As the initial pH increased, deprotonation started and the metal ions were complexed with the Fe_3_O_4_/rGO composites, hence the removal percentage of the Hg ions was elevated. As can be seen from Figure S7d,f, the Hg ion removal efficiency decreased with an increase in initial Hg ion concentration from 5 µg/L to 15 µg/L. This fact might be caused by the adsorption capacity of the absorbent.

The optimization of the process variables was performed using the quadratic model to maximize the removal percentage of the low-concentration mercury by the Fe_3_O_4_/rGO composites from the aqueous solutions. The removal efficiency predicted by the RSM model was 92.33% under the optimum conditions (*T* = 25.0 °C, initial pH = 10.0, *C* = 5.0 µg/L and *t* = 50.0 min). The model F-value (54.44) and the *p*-value (0.001) showed that the model was significant for the removal of low-concentration mercury by the Fe_3_O_4_/rGO composites.

### 3.3. Optimization of the ANN Architecture

The best structure of the ANN was selected on the basis of the maximum *R*^2^ value and the lowest value of *MSE* for the test set [48]. In the network optimization, one to 10 of the neurons were applied in the hidden layer. Seven neurons in the hidden layer was found to be the most suitable structure to best represent the Hg removal, as shown in Figure S8, therefore the ANN containing seven hidden neurons was selected as the suitable model with a value of 0.0066 for *MSE*. As shown in Figure S9, the *MSE* versus the number of epochs for an optimal ANN model indicates that the training was stopped after 1999 epochs. The predicted percentages of normalized Hg ion removal for the training set using the ANN model are plotted versus their experimental data (Figure S10), and the obtained coefficient of determination *R*^2^ was 0.9928 for the training set.

### 3.4. Optimization by the GA technique

**Figure 3:**
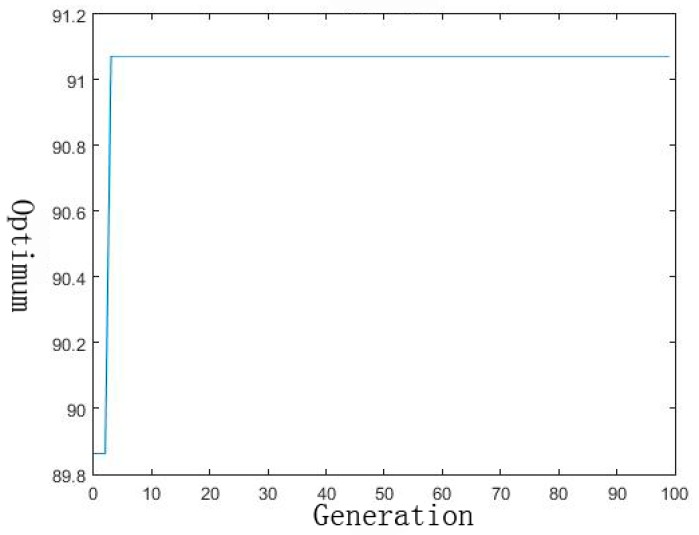
Evolution of fitness with 100 generations.

The developed ANN model was used for the optimization by the GA technique with the objective of maximizing the low-concentration mercury removal percentage from the aqueous solutions. The values of the GA-specific parameters used in the optimization technique were as follows: population size = 10, crossover probability = 0.8 and mutation probability = 0.01. Optimum conditions were selected by the evaluation of GA for 100 iterations to achieve a good percentage of low-concentration mercury removal. The optimum conditions were obtained as follows: contact time of 63.5 min, initial pH of 9.9, temperature of 37.3 °C and initial Hg ions concentration of 8.6 µg/L. The percentage of the Hg ion removal under these optimum conditions was 91.13%, predicted using the GA (Figure 3). The error between the predicted and experimental verification values was within 5%, which illustrates the validity of the constructed ANN-GA model.

### 3.5. Comparison of RSM Model with ANN-GA Model

In the present study, the results of these models (the RSM model and ANN-GA model) were compared and validated by confirmation experiments in the predicted optimal operating conditions (Table 1). The predicted value from the ANN-GA was closer to the experimental verification values and the *R*^2^ of ANN-GA (0.9928) was higher than that of the RSM (0.9007). This shows that the ANN-GA is more suitable for the optimization of the low-concentration mercury removal process than RSM in this study.

### 3.6. Adsorption Equilibrium Study

The equilibrium adsorption isotherm is used to provide useful information about the mechanism, properties and tendency of the adsorbent for low-concentration mercury in aqueous solutions, therefore it is vital to establish the most appropriate correlation for the equilibrium curve. The adsorption data were fitted to the Langmiur and Freundlich isotherm models [52].

The nonlinear and linear expressions of the Langmiur isotherm are expressed as follows:

(8) $$q_{e}=\frac{q_{m}k_{L}C_{e}}{1+k_{L}C_{e}}$$

(9) $$\frac{C_{e}}{q_{e}}=\frac{1}{k_{L}q_{m}}+\frac{C_{e}}{q_{m}}$$

where *C_e_* is the equilibrium concentration of metal ions (µg/L) in the solution, *q_e_* is the equilibrium adsorption capacity, *k_L_* is the Langmiur constant related to the adsorption energy and *q_m_* is the maximum adsorption capacity of the Fe_3_O_4_/rGO composites for monolayer coverage. The *q_m_* and *k_L_* values were obtained from the slope and intercept of the linear plot of *C_e_* versus *C_e_/q_e_*.

The nonlinear and linear expressions of the Freundlich isotherm are expressed as:

(10) $$q_{e}=k_{F}{\left(C_{e}\right)}^{1/n}$$

(11) $$\ln q_{e}=\ln k_{F}+\frac{1}{n}\ln C_{e}$$

where *k_F_* is the Freundlich constant related to adsorption capacity, and *n* is the heterogeneity factor which is related to the capacity and intensity of adsorption. *k_F_* and 1*/n* values are determined experimentally from the slope and intercept of the linear plot of log *q_e_* versus log *C_e_*. The Langmuir adsorption isotherm is based on the assumption that monolayer adsorption takes place on a homogeneous surface, while the Freundlich isotherm describes multilayer adsorption on a heterogeneous surface.

**Figure 4:**
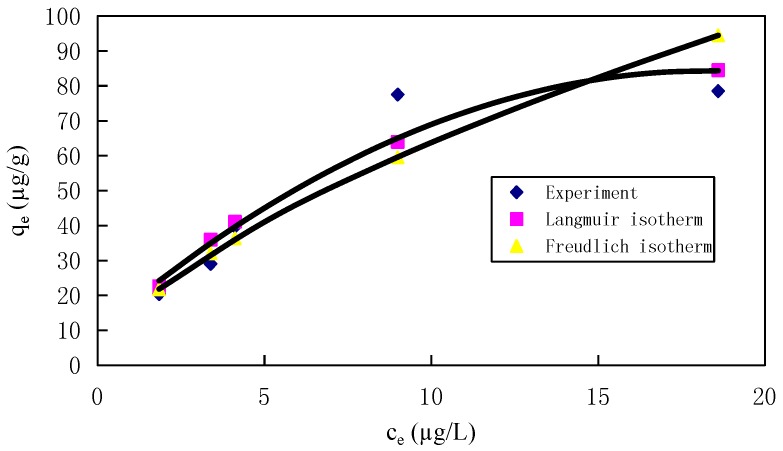
Experimental equilibrium data and isotherm obtained by the nonlinear regression method for the sorption of Hg ions on the Fe_3_O_4_/rGO composite (initial pH = 7.0, the Fe_3_O_4_/rGO composite dosage = 20 mg, temperature = 25 °C and contact time = 60 min).

The Langmuir model indicates a monolayer coverage on the surface of the adsorbent, while the Freundlich model is indicative of the surface heterogeneity of the adsorbent [53,54]. In this study, the isotherm plots for the Hg ion adsorption are shown in Figure S11, and the regression coefficients (*R*^2^) of the Langmuir and the Freundlich models obtained by linear fitting were 0.9049 and 0.9118, and those obtained by nonlinear fitting were 0.8775 and 0.9118 (Table 2), respectively. These facts suggest that the Hg ions were adsorbed in the mode of heterogeneity on the surface of the Fe_3_O_4_/rGO composites. This indicates that the Freundlich isotherm represents a better fit with the experimental data and confirms the applicability of the Freundlich model in the present study for the low-concentration mercury removal process. The small value of 1/*n* (0.6336) from the nonlinear fitting, which was between zero and one, indicated the heterogeneity of the Fe_3_O_4_/rGO composites with an exponential distribution of the energy of surface active sites, while the small 1/*n* and the large *k_F_* value (14.8140 µg/g from the nonlinear fitting) showed that the Fe_3_O_4_/rGO composites had a satisfactory adsorption capacity for low-concentration mercury in aqueous solutions. The experimental data and the isotherm models obtained by the nonlinear regression method for the sorption of Hg ions on the Fe_3_O_4_/rGO composites is shown in Figure 4, which demonstrates that the removal quantity from the Freundlich isotherm is generally more agreeable with the experimental data than that from the Langmuir isotherm.

### 3.7. Kinetic Study

The effects of contact time on the removal of low-concentration mercury by the Fe_3_O_4_/rGO composites were investigated. To understand the removal mechanism and kinetics, the pseudo-first-order and the pseudo-second-order kinetic models were used to fit the experimental data. The pseudo-first-order kinetic model is generally represented by the following equation [50,55,56]:

(12) $$\ln(q_{e}-q_{t})=\ln q_{e}-k_{1}t$$

where *q_t_* (µg·g^−1^) is the removal quantity of the Hg ions at time *t* (min), and *k*_1_ (min^−1^) is the rate constant. The values were calculated from the slope of the plots of ln (*q_e_* − *q_t_*) versus *t*, as given in Table 3. The pseudo-second-order rate equation is expressed as follows [57,58]:

(13) $$\frac{t}{q_{t}}=\frac{1}{k_{2}{q_{e}}^{2}}+\frac{t}{q_{e}}$$

where *q_t_* is the removal quantity of the Hg ions at time *t* (µg·g^−1^). The values of the pseudo-second-order rate constant *k*_2_ was determined from the slope and intercept of the plot of *t/q_t_* versus *t*.

**Figure 5:**
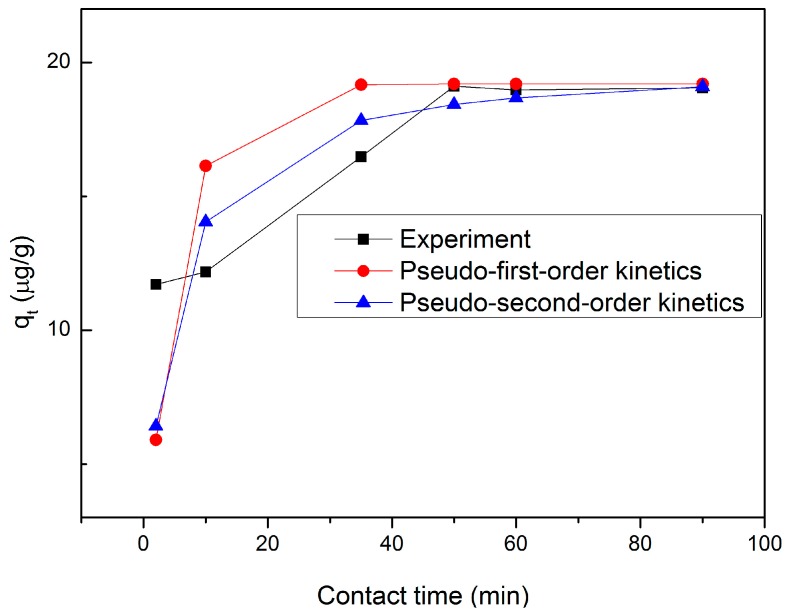
Time-dependent study of Hg ions removal by Fe_3_O_4_/rGO composites (initial pH = 7.0; Fe_3_O_4_/rGO dosage = 20 mg; temperature = 25 °C; Hg ions concentration = 10 µg/L).

The values of the corresponding model parameters are summarized in Table 3, which illustrates that the correlation coefficient for the pseudo-second-order kinetic model is greater than that for the pseudo-first-order kinetic model. This indicates that the removal system is best described by the pseudo-second-order kinetic model. The equilibrium was achieved for the full removal process within 60 min by the Fe_3_O_4_/rGO composites (Figure 5), and the removal quantity from the pseudo-second-order kinetic model is generally more agreeable with the experimental data than that from the pseudo-first-order kinetic model. The removal efficiency of low-concentration mercury by different adsorbents in a certain period of time is shown in Table 4, which demonstrates that the removal efficiency of the Fe_3_O_4_/rGO composites is higher than that of other materials.

### 3.8. The Low-Concentration Mercury Removal Mechanisms

The mechanism of any removal process may be governed by either physical, chemical or a combination of both processes [61]. The chemical process refers to the chemical interactions between the adsorbate and adsorbent that can be sub-divided into the chemisorption and physisorption interactions. The chemisorption interaction, such as ion-exchange, chelation and metal complex, is identified as a strong interaction, while the physisorption interaction, such as van der Waals interaction and H-bonding, is a weak interaction. The physical process includes the diffusion in bulk of liquid phase, diffusion through boundary layer and diffusion in micropores or mesopores, which co-exists with the chemical process during the entire period of the removal process. All these processes determine the mechanism of any removal process that depends on several factors, including the properties and nature of the adsorbent, chemical nature of adsorbate and removal process parameters [61]. The removal of the low-concentration mercury by the Fe_3_O_4_/rGO composites may be attributed to the variety of active groups present on their surfaces. The presence of oxygen active sites might come from the hydroxyl and carbonyl groups from the Fe_3_O_4_/rGO composites, which may be considered as either physisorption or chemisorption.

**Figure 6:**
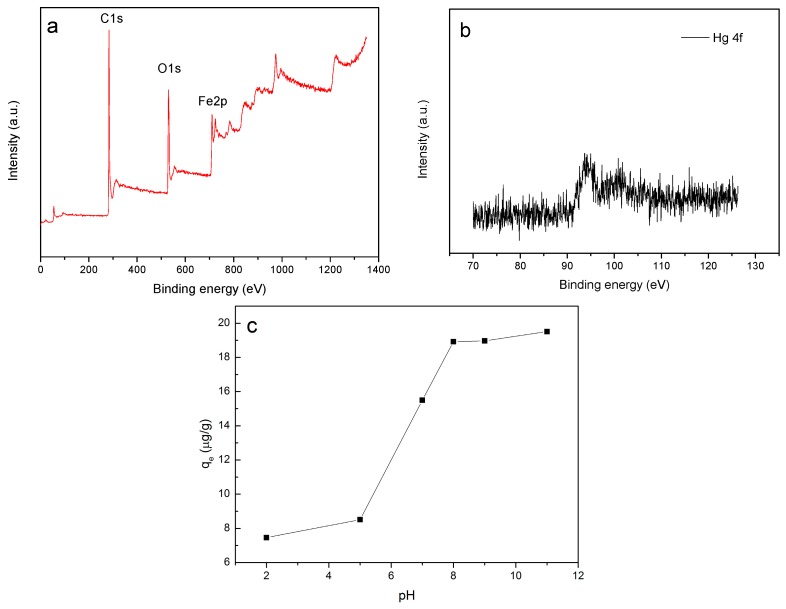
X-ray photoelectron spectroscopy(XPS) spectrum of wide scan for the Fe_3_O_4_/rGO composites after removal (**a**) and XPS spectrum of the Hg ions adsorbed onto the Fe_3_O_4_/rGO composites after removal (**b**); pH dependent adsorption of Hg ions on Fe_3_O_4_/rGO composites (Fe_3_O_4_/rGO dosage = 20 mg, temperature = 25 °C, Hg ions concentration = 10 µg/L) (**c**).

To further understand the removal processes for the low-concentration mercury, the Fe_3_O_4_/rGO composites after the Hg ion removal at an initial pH of around 7 for 70 min was analyzed with X-ray photoelectron spectroscopy (XPS). On the basis of the XPS study, a possible schematic mechanism can be proposed for mercury absorption by the Fe_3_O_4_/rGO composites. The peaks of Fe2p_3/2_, Fe2p_1/2_, O1s and C1s were found in the XPS spectrum of wide scan for the Fe_3_O_4_/rGO composites after the Hg ion removal (Figure 6a). This showed that Fe_3_O_4_ was successfully embedded in the rGO surface and that the Fe_3_O_4_/rGO composites possess a reasonable degree of stability. The XPS spectrum of Hg 4f for the Fe_3_O_4_/rGO composites after the Hg ion removal is shown in Figure 6b, which clearly confirms the removal of the Hg ions by the Fe_3_O_4_/rGO composites. The value of the binding energy for Hg 4f clearly indicates that Hg(0) (binding energy between 99.2 eV and 99.8 eV) was not present at the Fe_3_O_4_/rGO composite surface, thus the oxidation-reduction mechanisms can be excluded.

The pH of the solution influences the surface chemical properties of an adsorbent and the solution chemistry of the adsorbate in the aqueous solution. [62,63]. As shown in Figure 6c, the adsorption capacity increases with the increase of pH. At a low solution pH, the functional groups on the Fe_3_O_4_/rGO composite surface (i.e., hybrid–OH and hybrid–COOH) are protonated, forming a positive surface charge. A bond may be established between the H_3_O^+^ ions and the π-electron cloud of rGO [63]. The electrostatic repulsion between the positively charged adsorbent surface and the free Hg ions leads to competition between H^+^ and free Hg ions for the limited active surface [62]. When the solution pH increases, the concentration of H^+^ ions decreases, hence reducing the electrostatic repulsion and the competitive adsorption for higher Hg ion retention.

### 3.9. Regeneration and Stability of the Fe_3_O_4_/rGO Composites

**Figure 7:**
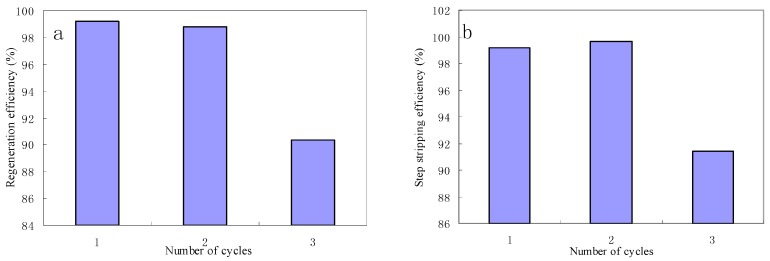
Evolution of regeneration efficiency (**a**) and the step-stripping efficiency (**b**) after each regeneration cycle (Fe_3_O_4_/rGO dosage = 20 mg, temperature = 25 °C, Hg ions concentration = 100 µg/L, pH = 7).

The stability of the adsorbent was verified in the regeneration process by using dilute acid. A total of 0.01 mol/L HCl was used for this regeneration process. The Fe_3_O_4_/rGO composites showed a high stability under the acidic condition. The composites containing mercury were recovered satisfactorily using 0.01 M HCl. As shown in Figure 7, the regeneration efficiency of the first cycle, second cycle and third cycle is 99.17%, 98.80% and 90.32%, respectively. This indicated that the regeneration efficiency gradually decreases with the subsequent cycle and the decrease is less than 10%. The step-stripping efficiency for the second cycle is slightly higher than for the first cycle, and with the third cycle begins to decrease obviously, thus the composites can be reused for the removal of low-concentration mercury from aqueous solutions.

## 4. Conclusions

In the present study, the Fe_3_O_4_/rGO composites were prepared by the solvothermal method and characterized by XRD, TEM, AFM, N_2_-sorption, XPS, FTIR and SQUID. RSM and ANN-GA were employed to model the effects of different operating conditions on the removal of low-concentration mercury from aqueous solutions by the Fe_3_O_4_/rGO composites. The removal efficiency predicted by the ANN-GA model was 91.10% in comparison with 86.72% from the confirmation experiment under the optimum conditions (*T* = 37.3 °C, initial pH = 9.9, *C* = 8.6 µg/L and *t* = 63.5 min), while the removal efficiency predicted by the RSM model was 92.33% in comparison with 82.67% from the confirmation experiment under the optimum conditions (*T* = 25.0 °C, initial pH = 10.0, *C* = 5.0 µg/L and *t* = 50.0 min). The ANN-GA model results (with a prediction error below 5%) showed better agreement with the experimental data than the RSM model results (with a prediction error below 10%), thus the validity of ANN-GA model was clearly confirmed. The removal results of low-concentration mercury could be well-fitted to the Freundlich isotherm, and the removal kinetics were well-described by the pseudo-second order kinetic model. In addition, the regeneration experiment of the Fe_3_O_4_/rGO composites demonstrated that these composites can be reused for the removal of low-concentration mercury from aqueous solutions. Therefore, Fe_3_O_4_/rGO composites are suited for the advanced treatment of wastewater with a low concentration of mercury. In order to facilitate practical applications of this technology, future studies should use a permeable reactive barrier (PRB) system to investigate low-concentration mercury removal from aqueous solutions with the aid of advanced AI tools.

## Figures and Tables

**Table 1:** Comparative results for optimization by artificial neural networks-genetic algorithm (ANN-GA) and response surface methodology (RSM).

Process Variable	Optimization	
	ANN-GA Optimization	
	RSM	ANN-GA
Initial pH	10.0	9.9
Initial Hg ions concentration (mg/L)	5.0	8.6
Temperature (°C)	25.0	37.3
Contact time (min)	50.0	63.5
Removal efficiency of model (%)	92.33	91.13
Experimental verification values (%)	82.67	86.72
*R*^2^	0.9007	0.9928

**Table 2:** The Langmuir and Freundlich isotherm parameters for the removal of the Hg ions by the Fe_3_O_4_/rGO composites.

Isotherms	Parameters	Value of Parameters Obtained by the Linear Fitting	Value of Parameters Obtained by the Nonlinear Fitting
Langmuir	*k_L_* (L/µg)	0.1159	0.1253
-	*q_m_* (µg/g)	120.4819	120.7952
-	*R*^2^	0.9049	0.8775
Freundlich	*k_F_* (µg/g)	7.3274	14.8140
-	1*/n*	0.6338	0.6338
-	*R*^2^	0.9118	0.9118

**Table 3:** Kinetic parameters for the removal of the Hg ions by the Fe_3_O_4_/rGO composites.

Model	Parameters	Value of Parameters
pseudo-first-order	*k*_1_ (1/min)	0.1836
-	*R*^2^	0.8102
-	*q_e_*	19.20
pseudo-second-order	*k*_2_ (g/mg·min)	0.0118
-	*R*^2^	0.9948
-	*q_e_*	19.84

**Table 4:** The low-concentration mercury removal efficiency by Fe_3_O_4_/rGO composites and other materials.

Materials	Adsorbent Dosage	Initial Concentration	Volume of Solution	Contact Time	Removal Efficiency	Reference
Fe_3_O_4_/SiO_2_/NH/CS_2_	3 mg	50 ppb	-	48 h	74.00%	[59]
Fe_3_O_4_/SiO_2_	3 mg	50 ppb	-	48 h	24.00%	
MOF	2 mg	1 ppb	40 mL	1 h	42.60%	[60]
	2 mg	2 ppb	40 mL	1 h	66.50%	
	2 mg	5 ppb	40 mL	1 h	70.98%	
	2 mg	10 ppb	40 mL	1 h	83.53%	
	2 mg	20 ppb	40 mL	1 h	84.76%	
Fe_3_O_4_/rGO	20 mg	8.6 ppb	50 mL	63.5 min	86.72%	Present study

## Supplementary Materials

The following are available online at www.mdpi.com/1996-1944/10/11/1279/s1, Table S1: The experimental domain factor and level for the Box-Behnken design, Table S2: Size distributions calculated from TEM images of Fe_3_O_4_/rGO composites, Table S3: The contents of different elements in the Fe_3_O_4_/rGO composites, Figure S1: XRD patterns of the GO (a), the Fe_3_O_4_/rGO composites (b) and the Fe_3_O_4_/GO composites (c), Figure S2: TEM image of the Fe_3_O_4_/rGO composites (a), 3D AFM topography image of the Fe_3_O_4_/rGO composites (b), vertical profile of the Fe_3_O_4_/rGO composites (c) and TEM image of the Fe_3_O_4_/GO composites (d), Figure S3: Adsorption/desorption isotherms of the Fe_3_O_4_/rGO composites (a) and BJH pore-size distribution curves of the Fe_3_O_4_/rGO composites (b), Figure S4: XPS spectra of the Fe_3_O_4_/rGO composites (a); the high-resolution spectra of Fe2p (b); C1s XPS spectra of the Fe_3_O_4_/rGO composites (c); O1s XPS spectra of the Fe_3_O_4_/rGO composites (d); XPS spectra of the Fe_3_O_4_/GO composites (e), Figure S5: FTIR spectra of the GO and the Fe_3_O_4_/rGO composites, Figure S6: The magnetization hysteresis loop of the Fe_3_O_4_/rGO composites (a); The magnetization hysteresis loop of the Fe_3_O_4_/GO composites (b), Figure S7: 3D surface plots for interactive effect of temperature and initial pH (a); temperature and contact time (b); temperature and initial Hg ions concentration (c); initial Hg ions concentration and contact time (d); initial pH and contact time (e); and initial pH and initial Hg ions concentration (f) on the removal of the Hg ions, Figure S8: Relationship between number of neurons and MSE, Figure S9: MSE versus the number of epochs, Figure S10: The experimental data versus the predicted data of normalized removal, Figure S11: Adsorption isotherm of the Hg ions by the Fe_3_O_4_/rGO composites (initial pH = 7.0; the Fe_3_O_4_/rGO composites dosage = 20 mg; temperature = 25 °C and contact time = 60 min).
