# Supplementary material for: Optimizing Low-Concentration Mercury Removal from Aqueous Solutions by Reduced Graphene Oxide-Supported Fe3O4 Composites with the Aid of an Artificial Neural Network and Genetic Algorithm
Source: Materials (Basel). 2017 Nov 7;10(11):1279. doi: 10.3390/ma10111279 (PMC5706226; doi:10.3390/ma10111279)
Supplement: Supplementary file 1 [file materials-10-01279-s001.pdf]

materials

MDPI

Article

Supplementary Materials

Optimizing the Low-Concentration Mercury Removal from Aqueous Solutions by Reduced Graphene Oxide-supported Fe_3O_4 Composites with the Aid of Artificial Neural Network-genetic Algorithm

The graphene oxide-supported Fe_3O_4 (Fe_3O_4/GO) composites was synthesized by the in situ chemical co-precipitation of Fe^{2+} and Fe^{3+} in an alkaline solution in the presence of GO [1]. Firstly, 100 mL GO (3 mg·mL^{-1}) aqueous solution was sonicated for 1 h. 2.7030 g of $FeCl_3 \cdot 6H_2O$ and 1.9881 g of $FeCl_2 \cdot 4H_2O$ were dissolved in 10 mL of distilled water and which was purged with N_2 for 30 min. This solution was added dropwise to the GO solution at room temperature under a nitrogen flow with vigorous stirring for 2 h. Then the resulting mixture was heated to 65 °C before ammonia solution was added to adjust the pH to 10. The mixture was kept stirring at 65 °C for a further 2 h. The precipitate was washed with ethanol and deionized water several times, and finally dried at 40 °C in a vacuum oven.

Table S1. The experimental domain factor and level for the Box-Behnken design.

Factors	Name	Units	Range and levels (coded)		
			−1	0	1
X_1	Temperature	°C	25	35	45
X_2	Time	min	50	60	70
X_3	Initial Hg ions concentration	μg/L	5	10	15
X_4	Initial pH	-	8	9	10

Table S2. Size distributions calculated from TEM images of Fe_3O_4/rGO composites.

Distridution/nm	Mean/nm	Amount	Freqency
171-199.2	185.1	2	8.70%
199.2-227.4	213.3	1	4.35%
227.4-255.6	241.5	1	4.35%
255.6-283.8	269.7	3	13.04%
283.8-312	297.9	1	4.35%
312-340.2	326.1	4	17.39%
340.2-368.4	354.3	0	0.00%
368.4-396.6	382.5	3	13.04%
396.6-424.8	410.7	2	8.70%
424.8-453	438.9	6	26.09%

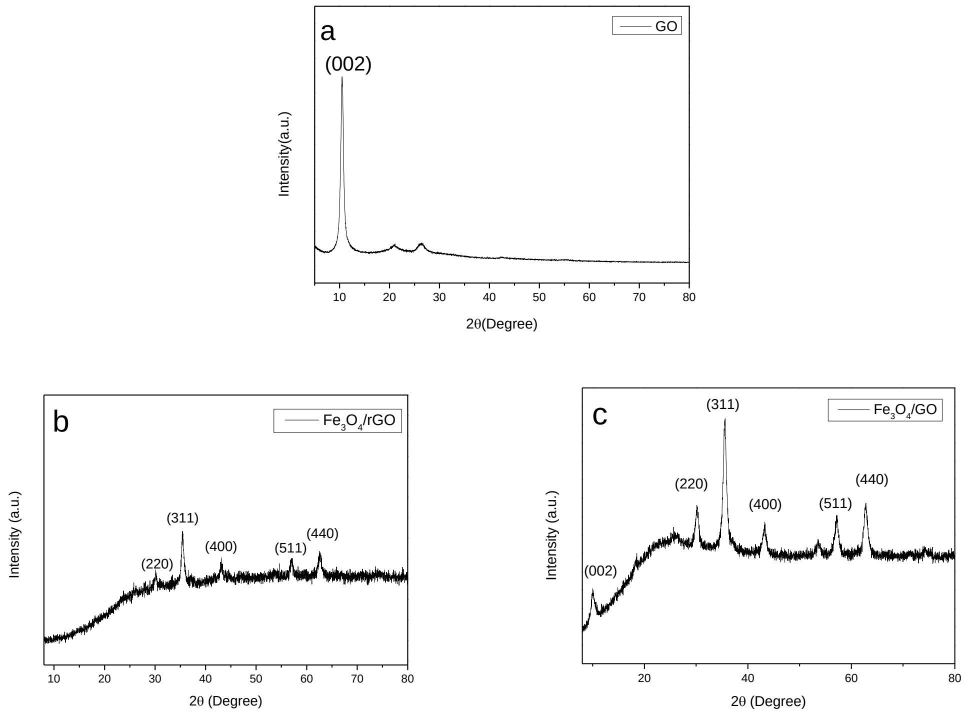

Figure S1. XRD patterns of the GO (a), the Fe_3O_4/rGO composites (b) and the Fe_3O_4/GO composites (c).

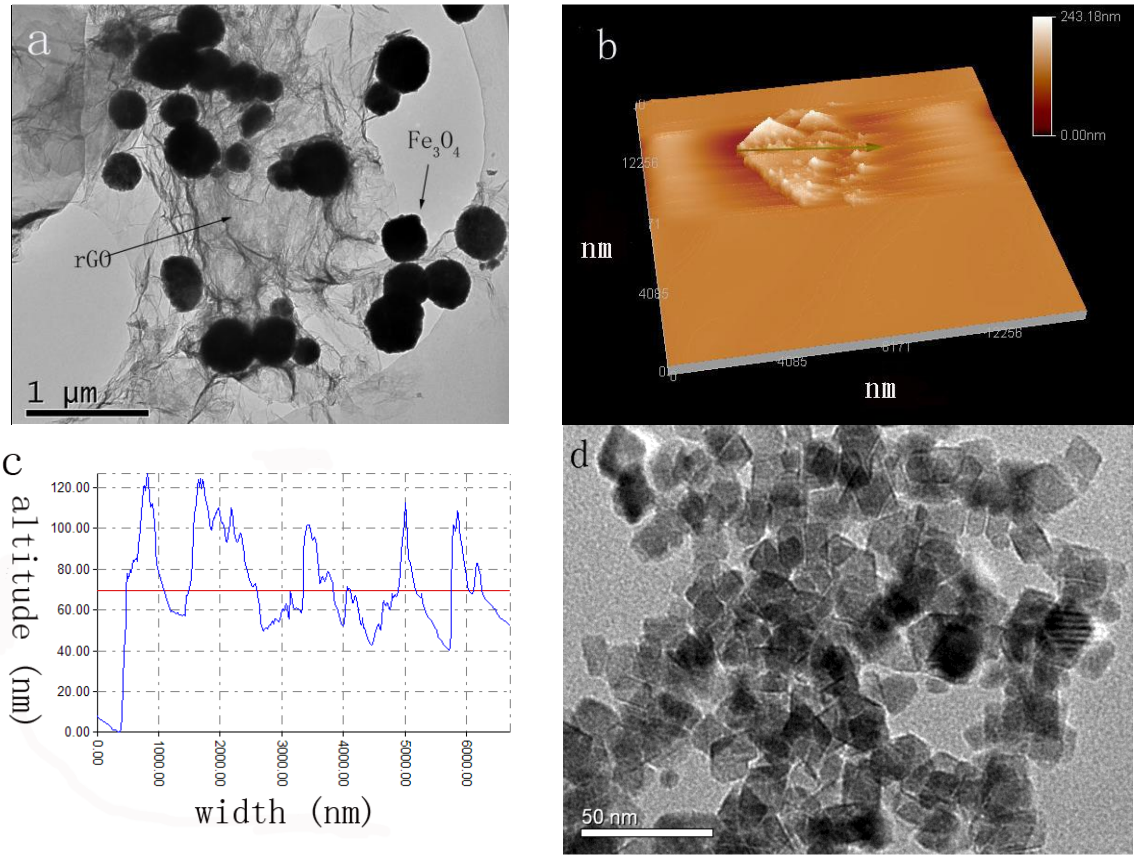

Figure S2. TEM image of the Fe_3O_4/rGO composites (a), 3D AFM topography image of the Fe_3O_4/rGO composites (b), vertical profile of the Fe_3O_4/rGO composites (c) and TEM image of the Fe_3O_4/GO composites (d).

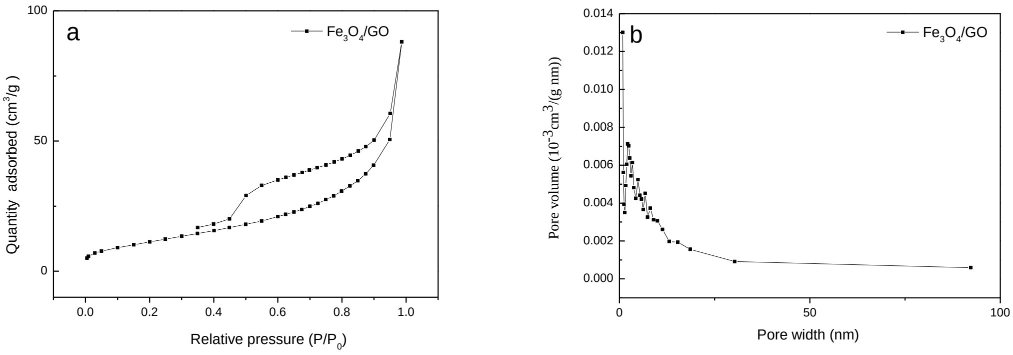

Figure S3. Adsorption/desorption isotherms of the Fe_3O_4/rGO composites (a) and BJH pore-size distribution curves of the Fe_3O_4/rGO composites (b).

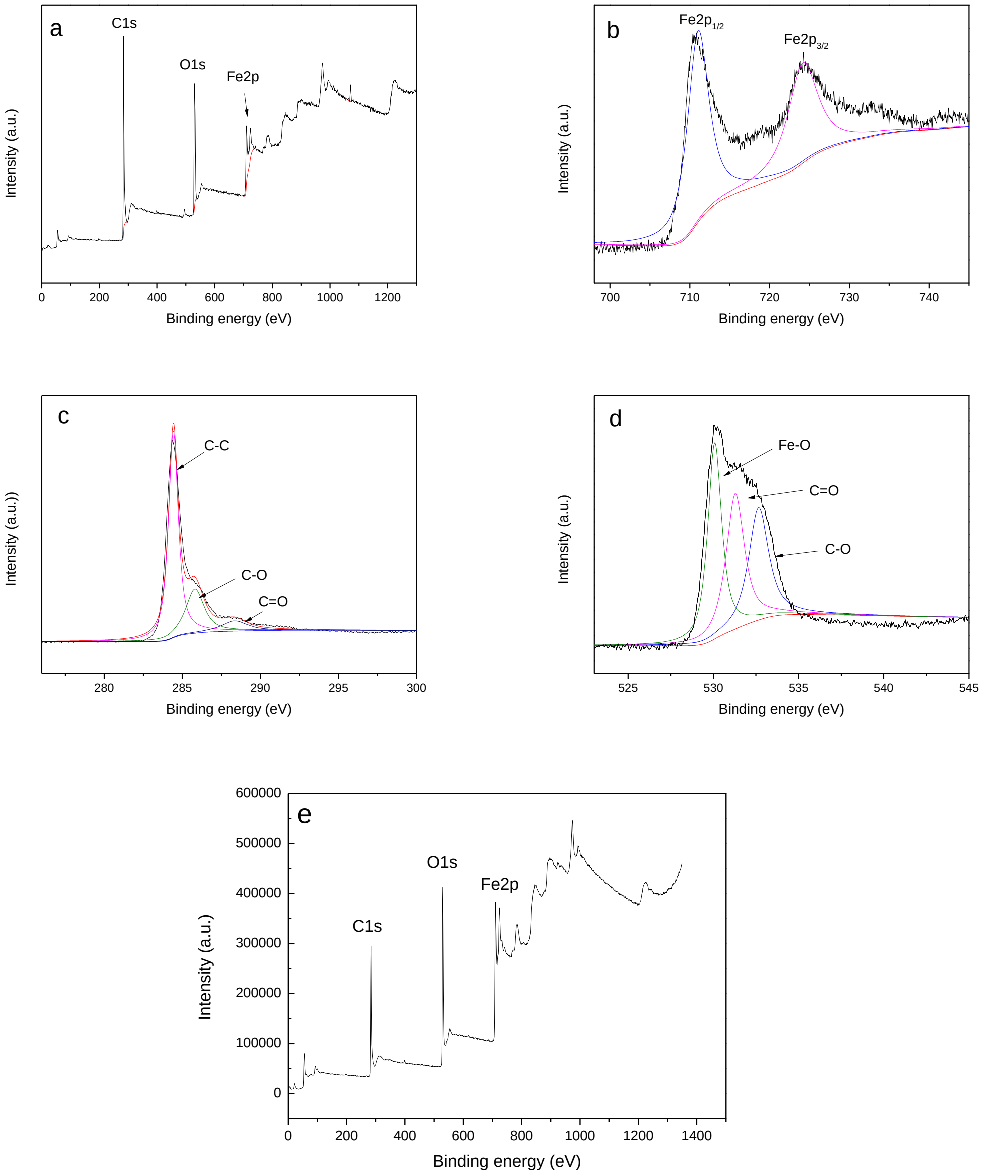

Figure S4. XPS spectra of the Fe_3O_4/rGO composites (a); the high-resolution spectra of Fe2p (b); C1s XPS spectra of the Fe_3O_4/rGO composites (c); O1s XPS spectra of the Fe_3O_4/rGO composites(d); XPS spectra of the Fe_3O_4/GO composites (e).

Table S3. The contents of different elements in the Fe_3O_4/rGO composites.

Name	C	O	Fe
content in the adsorbent (%)	68.049	24.66	7.291

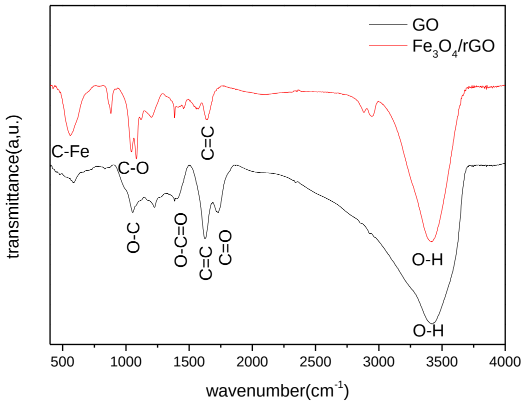

Figure S5. FTIR spectra of the GO and the Fe_3O_4/rGO composites.

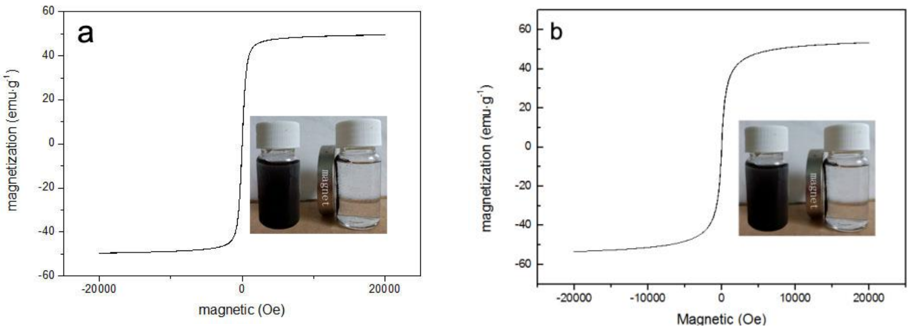

Figure S6. The magnetization hysteresis loop of the Fe_3O_4/rGO composites (a); The magnetization hysteresis loop of the Fe_3O_4/GO composites (b).

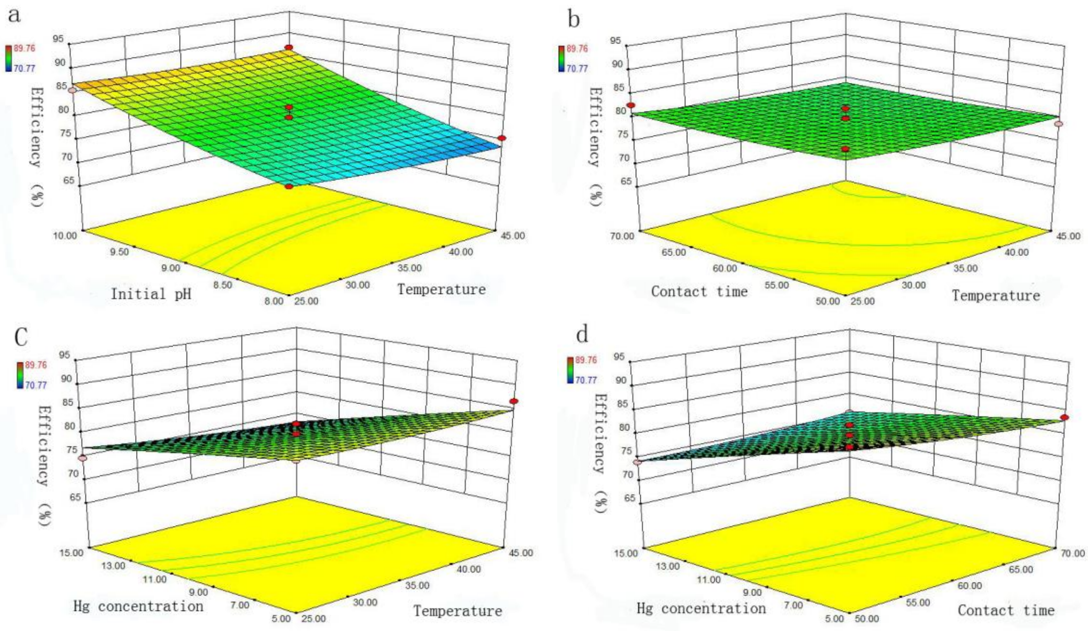

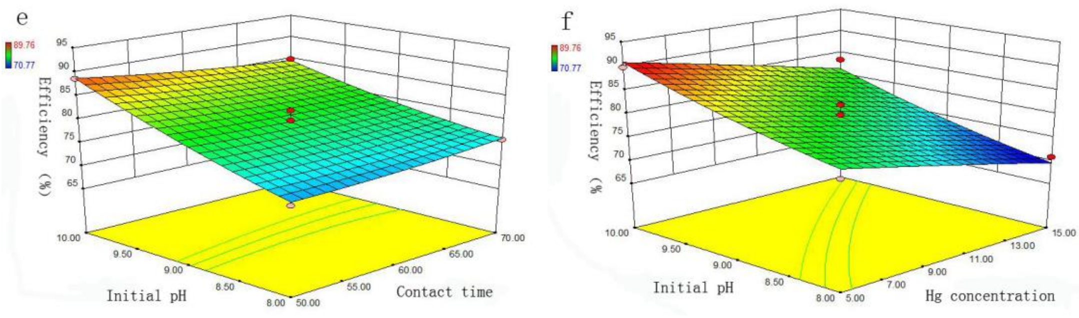

Figure S7. 3D surface plots for interactive effect of temperature and initial pH (a); temperature and contact time (b); temperature and initial Hg ions concentration (c); initial Hg ions concentration and contact time (d); initial pH and contact time (e); and initial pH and initial Hg ions concentration (f) on the removal of the Hg ions.

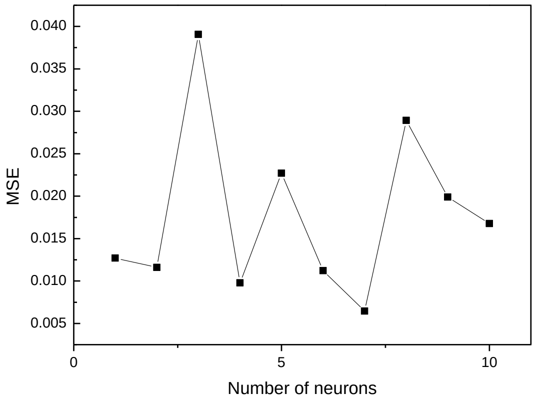

Figure S8. Relationship between number of neurons and MSE.

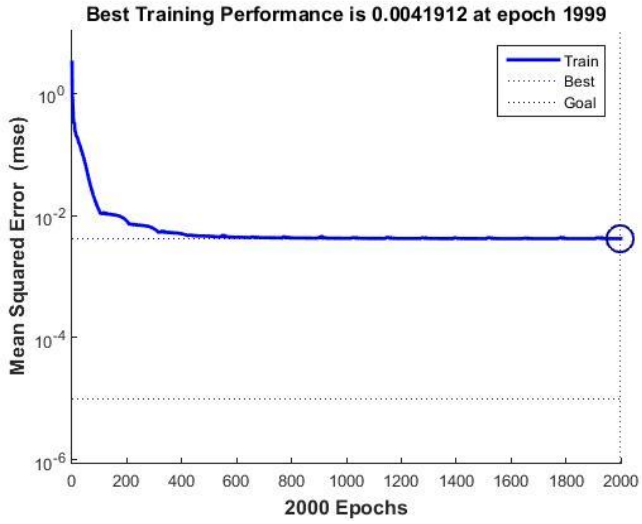

Figure S9. MSE versus the number of epochs.

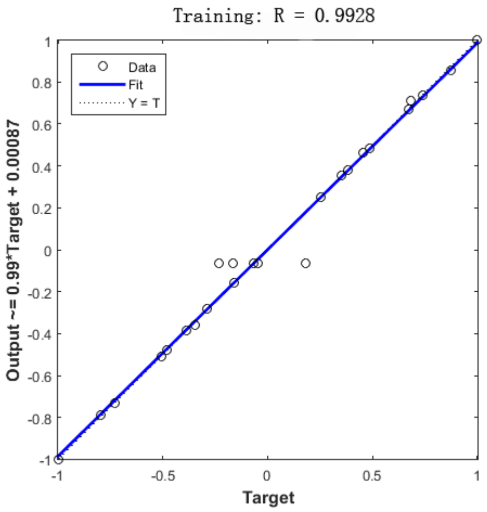

Figure S10. The experimental data versus the predicted data of normalized removal.

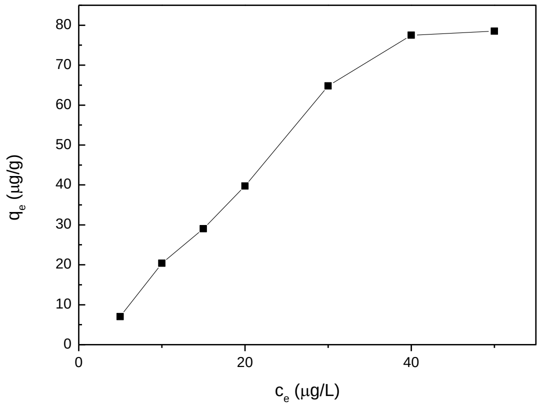

Figure S11. Adsorption isotherm of the Hg ions by the Fe_3O_4/rGO composites (initial pH = 7.0; the Fe_3O_4/rGO composites dosage = 20 mg; temperature = 25 °C; and contact time = 60 min).

References

1. Zeng, S.; Gan, N.; Weideman-Mera, R.; Cao, Y.; Li, T.; Sang, W. Enrichment of polychlorinated biphenyl 28 from aqueous solutions using Fe_3O_4 grafted graphene oxide. *Chem. Eng. J.* **2013**, *218*, 108–115.

© 2017 by the authors. Submitted for possible open access publication under the terms and conditions of the Creative Commons Attribution (CC-BY) license (http://creativecommons.org/licenses/by/4.0/).
